# Supplementary material for: Cardiologist-level interpretable knowledge-fused deep neural network for automatic arrhythmia diagnosis
Source: Commun Med (Lond). 2024 Feb 28;4:31. doi: 10.1038/s43856-024-00464-4 (PMC10901870; doi:10.1038/s43856-024-00464-4)
Supplement: Supplementary file 2 — Supplementary information [file 43856_2024_464_MOESM2_ESM.pdf]

Supplementary Information

Supplementary Methods

Data pre-processing

In the process of ECG recordings acquisition, technicians or residents prolong the sampling duration properly when abnormal heartbeats occur. Therefore, the under-sampling technique is implemented to handle varied input problem. According to the existing articles[1,2], under-sampling technique is a common and effective pre-processing method in the ECG classification field. Notably, we resample each recording $x_i^{t_i}$ to keep each input $\tilde{x}_i^{t_i}$ equal in length of 4096 sampling points, which does not change the diagnosis of the disease under study. For example, the critical features required for the atrial fibrillation and premature beats diagnosis, such as the RR intervals variability, remain unchanged before and after under-sampling. And then, under-sampling can improve model performance, and other methods like padding or truncation may cause the loss of signal information and consequently limit model performance. Besides, only one ECG recording is retained mostly for each person for the reason of signal specificity. Supplementary Figure 3 shows the input signals formed after under-sampling of ECG signals with different lengths.

The architecture of SJTU-ECGNet

Supplementary Figure 4 shows the architecture of the proposed SJTU-ECGNet, which contains multi-scale feature extractor, ATT layer, multi-label classifier and medical post-processing module.

CNN and Residual block

Fukushima[3] and Lecun et al.[4] first proposed and further explored the CNN applications, which have strong representation capability and have been utilized in different fields, such as mechanical metamaterial design[5], clinical diagnosis systems[6, 7], and all-cause mortality prediction[8].

Recently, some researchers[9] proposed an opinion that the increasing of CNN layers cannot continuously improve the model performance but reduce the model performance through experiments. Inspired by the thought of the article[9], we use residual blocks in our DL-based module. Equation (1)-(2) show the basic process of residual learning. Input vectors are signified as X, the nonlinear mapping function is signified as *F*(.) and the linear shortcut function is defined as *H*(.). Equation (1) represents the residual learning process in which the input dimension is consistent with the output dimension. And Equation (2) represents the residual learning process in which the input dimension is inconsistent with the output dimension.

$$Y_{L,1} = F\left(X_L, W_L, b_L\right) + X_L \tag{1}$$

$$Y_{L,2} = F\left(X_L, W_L, b_L\right) + H\left(X_L\right) \tag{2}$$

To sum up, we combine the advantages of CNN and Residual learning to design different Residual blocks for modelling ECG detection system, which is described in Supplementary Figure 5.

BiLSTM

As for processing time series[10], LSTM utilizes various gates for controlling information flow, which can acquire short-term and long-term features. Supplementary Figure 6 describes the details of LSTM architecture used in this paper.

$$f_t = \sigma\left(W_f[h_{t-1}, x_t^i] + b_f\right) \tag{3}$$

$$i_t = \sigma\left(W_i[h_{t-1}, x_t^i] + b_i\right) \tag{4}$$

$$o_t = \sigma\left(W_o[h_{t-1}, x_t^i] + b_o\right) \tag{5}$$

LSTM structure contains the forget gate (*f*), the input gate (*i*) and the output gate (*o*). Finally, the next output hidden state and the cell state are listed as follows.

$$\tilde{C}_t = \tanh\left(W_C[h_{t-1}, x_t^i] + b_C\right) \tag{6}$$

$$C_t = f_t \otimes C_{t-1} + i_t \otimes \tilde{C}_t \tag{7}$$

$$h_t = o_t \tanh\left(C_t\right) \tag{8}$$

Afterwards, LSTM updates weights by utilizing historical information on different gates. To better use forward and reverse history information, this paper uses BiLSTM for obtaining two-way features.

ATT layer

In the study of human vision, due to the limitation of information processing, humans selectively focus on a certain part of the input and ignore other information. The abovementioned mechanisms are referred to as attention mechanisms. The attention layer is a plug-and-play module that can theoretically be placed on each location to enhance beneficial features and show how much the model pays attention to the features.

Supplementary Figure 7 shows the ATT layer used in this paper. Firstly, the maximum and average feature values on the channel are taken from the input feature layer. Then, we stack these two results and use convolution layer to adjust the number of channels. After that, sigmoid function is used for activating the features. Meanwhile, in the input feature layer, the weight of each feature point is obtained (between 0-1). Finally, we multiply the original input feature layer with the obtained weights.

Multi-label classifier

As for multi-class arrhythmias classification, softmax classifier is a common classifier, and then, cross-entropy function is utilized as loss function.

$$p_{i,m} = \frac{e^{z_{i,m}}}{\sum_m e^{z_{i,m}}} \tag{9}$$

$$\mathrm{y}_i = \arg\max_m \left(p_{i,m} \right) \tag{10}$$

$$Loss(p_{i,m}) = -\alpha(1 - p_{i,m})^{\gamma} * \log\left(p_{i,m} \right) \tag{11}$$

Equation (9)-(10) represent softmax classifier and Equation (11) means the Focal loss function, where z_i denotes the input vector and y_i denotes the output. Because an ECG recording may have multiple arrhythmia diseases coupled, the classifier needs to be able to accurately separate each arrhythmia. Thus, we use the sigmoid function to design a multi-label classifier, which is described as follows.

$$\tilde{P}_{i,m} = \frac{1}{1+e^{-z_{i,m}}} \tag{12}$$

$$\tilde{y}_{i,m} = \begin{cases} 1, & if \quad \tilde{P}_{i,m} \geq threshold \\ 0, & if \quad \tilde{P}_{i,m} < threshold \end{cases} \tag{13}$$

Sigmoid function is applied to calculate each arrhythmia probability, which is listed in Equation (12)-(13). Supplementary Figure 8 shows the process of the proposed multi-label classifier. As stated in the literature reference[11], the commonly used fixed threshold for multi-label problems is 0.5. Therefore, we chose the threshold of 0.5 for our multi-label classifier.

Medical Post-processing Module

In clinical practice, the clinician's identification of any arrhythmia is accompanied by the strict medical criteria. And especially, the clinicians always need to calculate several important ECG parameters carefully such as heart rate, RR intervals to distinguish whether it's normal or not. However, it is not straightforward for the DL-based module to excavate the boundaries of these crucial parameters.

In detail, DL-based model encounters performance degradation and confusion when classifies the arrhythmias that require accurate parameters judgement. This is mainly because some common arrhythmia diseases, including ST and SB, depend on heart rate, RR interval and other indicators. These diagnostic indicators depend on the accurate position of the R-peaks. However, the DL-based model cannot accurately extract R-peaks, which will have a negative impact on the diagnostic effect of such arrhythmias.

Therefore, we propose a rule-based model from medical prior knowledge. In detail, firstly, we define the class set {y'} for this module only contains sinus rhythm recordings, namely Normal, ST, and SB. Then, for $x_i^{t_i}$, when the output $\tilde{y}_i \cap \left\{ y' \right\} \neq \varnothing$, we classify it using Supplementary Table 1.

In Supplementary Table 1, we conclude the medical post-processing algorithm of our model. Lines 1-3 initialize the final output and prepare two medical features for the rule models. Lines 4-6 show the ST and SB rule models where *index*$_{HR}$ can be calculated by Equation (14).

$$index_{HR} = \begin{cases} [0,1,0]^T, & if \quad HR > 100 \\ [1,0,0]^T, & if \quad 60 < HR \leq 100 \\ [0,0,1]^T, & if \quad HR \leq 60 \end{cases} \tag{14}$$

Performance metric

The important reason why the automatic diagnosis system is difficult to be applied in practice is the lack of a complete and standardized ECG dataset. Nowadays, ECG recordings are mainly stored in either image or PDF format, which is difficult to be applied in training AI models. And then, the open-source ECG datasets collect data on few patients, cover fewer types of arrhythmias, and have no complete diagnostic notes. Therefore, with the assistance of Shanghai First People's Hospital, this paper constructs LSCP-ECGDS to meet the requirements of out-of-hospital ECG diagnosis. Furthermore, we design different experiments to validate the feasibility of our approach.

In machine learning work, the precision (P) and recall (R) can be calculated by Equation (15) and Equation (16).

$$P = \frac{TP}{TP + FP} \tag{15}$$

$$R = \frac{TP}{TP + FN} \tag{16}$$

where TP=True Positive, FP=False Positive, TN=True Negative, FP=False Negative.

Accuracy is the ratio of the number of correctly classified samples to the total number of samples, which is signified as Equation (17)

$$Acc = \frac{TP + TN}{TP + TN + FP + FN} \tag{17}$$

Considering the generalizability of the model, F1 score is proposed with precision and recall, which is clarified in Equation (18). For the case of multiple classification, the overall performance of the model is evaluated by F1-macro in Equation (19), which is immune to data imbalance effects.

$$F1 = \frac{2 \times P \times R}{P + R} \tag{18}$$

$$F1 - macro = \frac{1}{n}\sum_{i=1}^{n} F_i \tag{19}$$

Where F_i denotes F1 score of the i class.

Supplementary Results

Diagnostic consistency

We choose **Hamming Loss** to express the diagnostic bias between any two objects. The values in each position of the matrix in Figure 2(a) are expressed as follows:

$$\mathrm{M}_{ij} = \frac{1}{mq}\sum_{k=1}^{m}\sum_{n=1}^{q} I\left(y_{i,n}^{(k)} \neq y_{j,n}^{(k)}\right) \tag{20}$$

Here, *i* and *j* represent the position in the matrix, *m* is the samples number and *q* is the classes number. For example, *M*(1,2) indicates the diagnostic bias between cardiologist1 and cardiologist2 while interpreting the ECGs. The lower the value, the more consistent the diagnosis between the two doctors. Through analysis, it can be determined that there is subtle inconsistency in diagnosis among cardiologists.

Supplementary Discussion

Comparison results of different segmentation algorithm

After conducting a thorough literature review, we utilize a recent deep learning-based R wave localization (RPNet) algorithm[12] to implement medical post-processing. We compare this method with the Hamilton segmentation algorithm and find that this approach is inferior to the Hamilton segmentation algorithm. The comparison results on the hidden set are listed as follows.

As shown in the Supplementary Table 2, we conduct an analysis on these two models, the Hamilton segmentation algorithm has a better performance than deep learning-based R waves location algorithm. What's more, we visualize two cases to show the differences between two algorithms. As shown in Supplementary Figure 10, the yellow line represents that RPNet ignores some R points during the point searching process, resulting in inaccurate RR feature calculation.

Generally, deep learning point searching algorithms do not exhibit border effects and have a wider range of point search. However, their algorithmic stability is inadequate. Therefore, the traditional Hamilton segmentation algorithm is more suitable for our research in this paper.

Comparison results of different cardiologists

And then, regarding the misinterpretation of ECG recordings, we conduct an analysis of all 292 ECGs that are not interpreted correctly by all of the three cardiologists. We then compare the diagnostic accuracy of the cardiologists and our model. The individual diagnostic accuracy of the three cardiologists is 37.33%, 18.49%, and 40.75%, respectively, whereas the model's accuracy is 80.48%. Based on these results, we conclude that our model performed better than the cardiologists in interpreting ECG recordings that are challenging for them. Therefore, we believe that the proposed method has important implications for clinical practice, particularly in cases where the accuracy of ECG interpretation is critical.

Additionally, we focus on comparing and analysing the diagnostic accuracy and F1-macro of our model with that of the cardiologists. As shown in the Supplementary Figure 11, We find that our model achieves an accuracy of 93.74%, which is higher than the accuracy of three of the cardiologists (81.13%, 86.28%, and 85.49%). Therefore, our model exhibits a cardiologist-level diagnostic accuracy on the hidden set. Regarding the F1-macro, we calculate it for both the cardiologists and the model. The experimental results indicate that the F1-macro of the cardiologists ranged from 79.40% to 92.88%, while our model achieves an F1-macro of 83.51%, which demonstrates a similar level of performance to that of the cardiologists.

Specifically, we calculate these metrics for both our model and the three cardiologists on the hidden set. As shown in the Supplementary Table 3, SJTU-ECGNet achieves better results in terms of specificity and NPV, while achieving comparable results with the cardiologists in terms of sensitivity and PPV.

Comparison results of different datasets

We test the generalization of the proposed model on the external Hospital dataset and use MIT-BIH[13] dataset, CODE[14, 15] dataset and PTB-XL[16] dataset as external datasets for evaluating the model generalizability of the proposed SJTU-ECGNet structure. The ECG recordings in last three datasets are obtained from clinical settings that are completely different from the

proposed dataset, with a high degree of independence. Supplementary Table 4 lists the experimental results.

As shown in the Supplementary Table 4, the proposed SJTU-ECGNet has good generalization, and also achieves 93.29% accuracy and 82.21% F1-macro on the external Hospital dataset, which is equivalent to the performance on the test set and hidden set, without overfitting. What's more, SJTU-ECGNet can also effectively extract the representative features from different datasets, and achieve 91.07%, 99.21%, 89.28% accuracy and 86.44%, 97.02%, 79.79% F1-macro in the last three datasets, respectively. According to the existing research, the accuracy of ECG judgment by general clinicians is about 80%. The experimental results show that SJTU-ECGNet can achieve good model performance in different datasets. Therefore, the proposed network structure has good generalization ability.

Training time consumption of SJTU-ECGNet

This paper trains the proposed SJTU-ECGNet structure on the local workstation with NVIDIA GTX 1080 GPU. Supplementary Table 5 shows the training cost of SJTU-ECGNet. During the training process, we set batch size as 128. According to the statistical results, each epoch of training takes 67.79s, that is, each batch takes 0.22s, and each ECG recording takes 0.0017s to train. In future work, we will configure workstations with better performance, which will greatly reduce the time-consuming of training the model. Meanwhile, the model can be used directly after training, which will ensure the real-time requirements of the model application.

Supplementary Table 1: Medical post-processing module algorithm

Supplementary Algorithm 1: medical post-processing module

Input: predicted sinus rhythm recording $x_i^{t_i}$; predicted label $\tilde{y}_i$

1. Initialize y_i with $\tilde{y}_i$.
2. Get R_i series using Hamilton segmentation algorithm[17]
3. Get *HR*, *RR intervals* features from R_i
4. ST and SB model:
5. Get $index_{HR}$ by Equation (14).
6. Update y_i using $index_{HR}$.

Output: the final output y_i

Supplementary Table 2: Comparison results of Hamilton segmentation and RPNet in the hidden set

	Normal	ST	SB	PAC	AF	PVC	Average
**Hamilton**	**97.32**	**94.18**	85.00	**56.41**	**82.93**	**85.25**	**83.51**
RPNet	85.89	92.63	85.00	56.41	82.93	85.25	80.44

Supplementary Table 3: Four metrics comparison results of SJTU-ECGNet and cardiologists on the hidden set

	Sensitivity	Specificity	PPV	NPV
**SJTU-ECGNet**	81.86	**98.50**	86.31	**98.52**
Cardiologist1	85.21	93.50	91.69	98.20
Cardiologist2	74.01	90.61	89.48	97.32
Cardiologist3	**92.97**	93.48	**93.01**	98.48

Supplementary Table 4: Comparison results of MIT-BIH[13], CODE[14, 15] and PTB-XL[16] and external Hospital datasets

	Normal	ST	SB	PAC	AF	PVC	F1-macro	Accuracy
External Hospital	96.80	86.54	85.65	69.21	79.04	76.00	82.21	93.29
MIT-BIH[13]	94.47	-	-	65.77	93.69	91.83	86.44	91.07
CODE[14, 15]	99.61	97.33	94.30	-	96.83	-	97.02	99.21
PTB-XL[16]	95.94	89.76	76.33	44.25	93.76	78.70	79.79	89.28

Supplementary Table 5: Training time consumption of SJTU-ECGNet

	one epoch	one batch	one ECG recording
Time consumption	67.79 s	0.22 s	0.0017 s

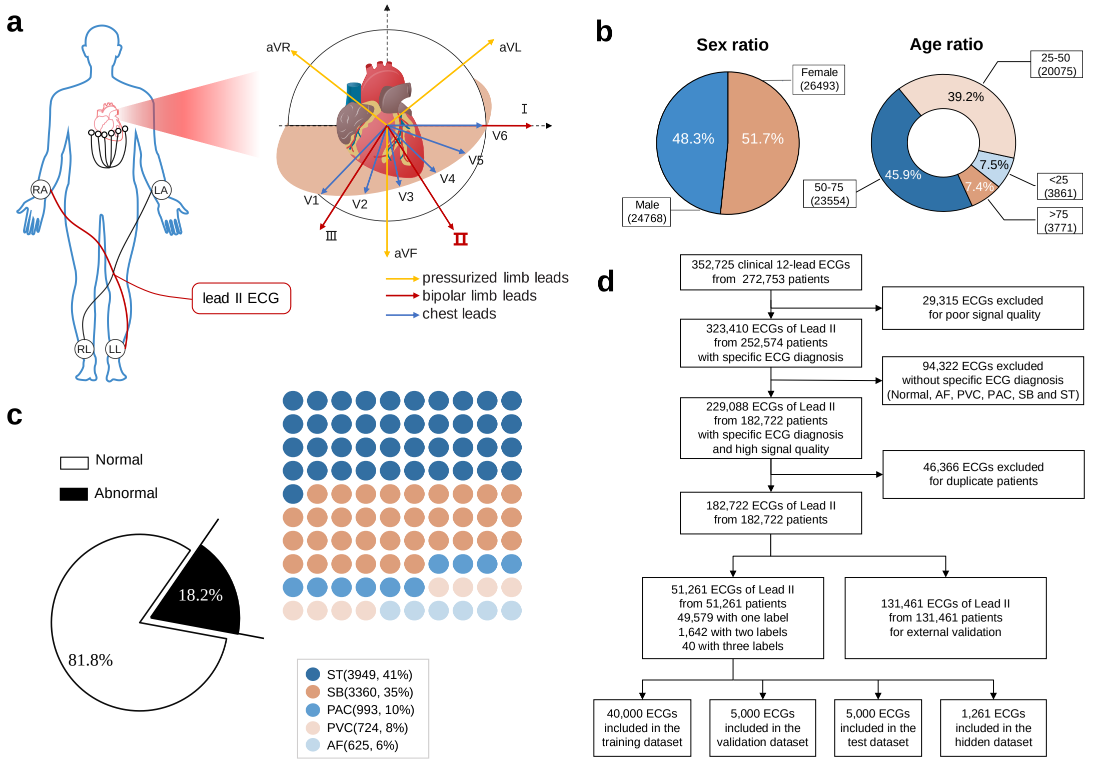

Supplementary Figure 1: Organization for Large-scale Chinese people's ECG dataset (LSCP-ECGDS). a The 12 leads ECG measurement system, including 6 limb leads (3 pressurized limb leads and 3 bipolar limb leads) and 6 chest leads. Lead II is adopted in this paper considering the convenience of wearing the sampling equipment. **b** Dataset distribution. The dataset comprises 51,261 clinical recordings from over 50,000 patients by carefully selecting from the whole LSCP-ECGDS, where 48.3% are male and 51.7% are female with ages covering the range from the young to the elder (7.5% below 25 years, 39.2% from 25 to 50 years, 45.9% from 50 to 75 years and 7.4% over 75 years). **c** The six common arrhythmias consist of 81.8% normal cases and 18.2% abnormal cases. Within the abnormal category, the following subcategories are present: ST (41%), SB (35%), PAC (10%), PVC (8%), and AF (6%). **d** STARD (Standards for Reporting of Diagnostic Accuracy Studies) flow diagram of LSCP-ECGDS.

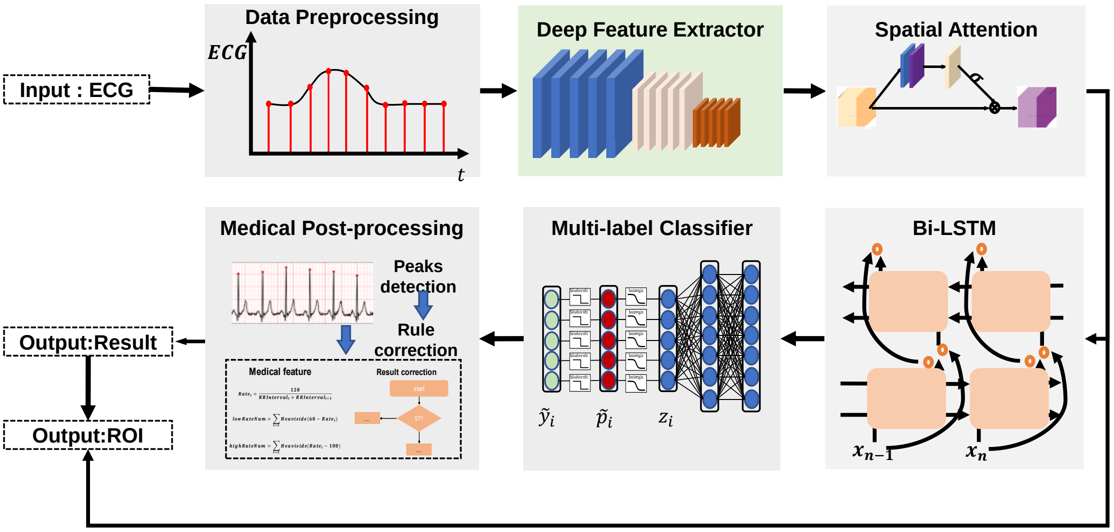

Supplementary Figure 2: Schematic diagram of the proposed deep-learning-based and medical-knowledge-involved arrhythmias classification method.

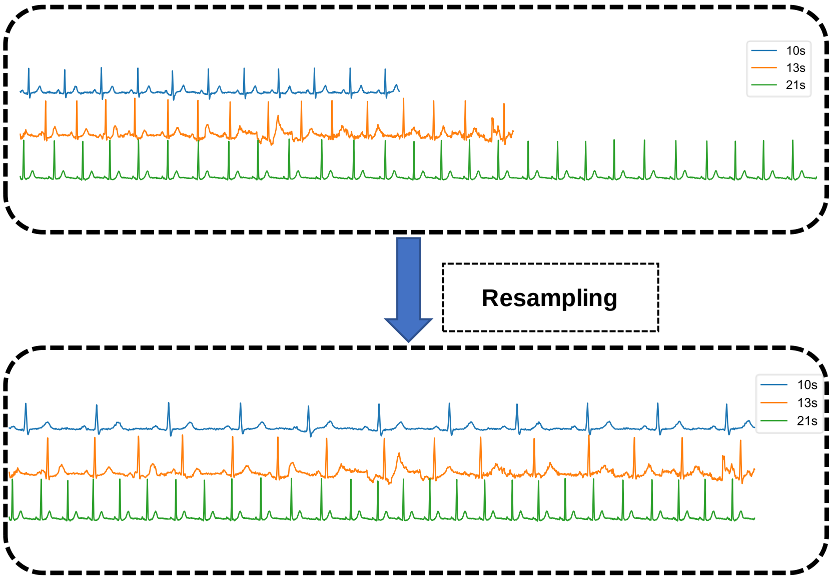

Supplementary Figure 3: Under-sampling process.

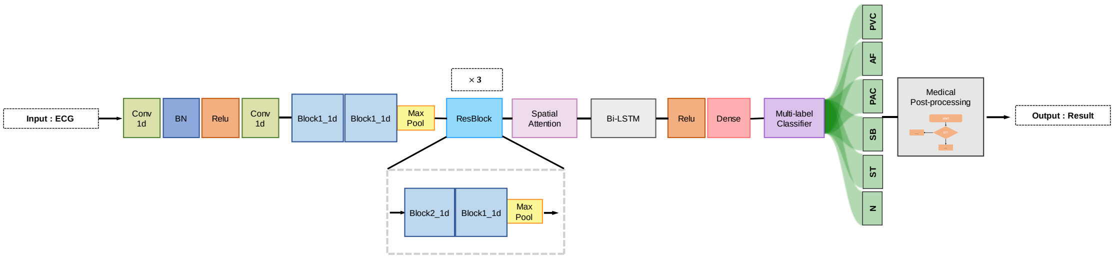

Supplementary Figure 4: The architecture of SJTU-ECGNet.

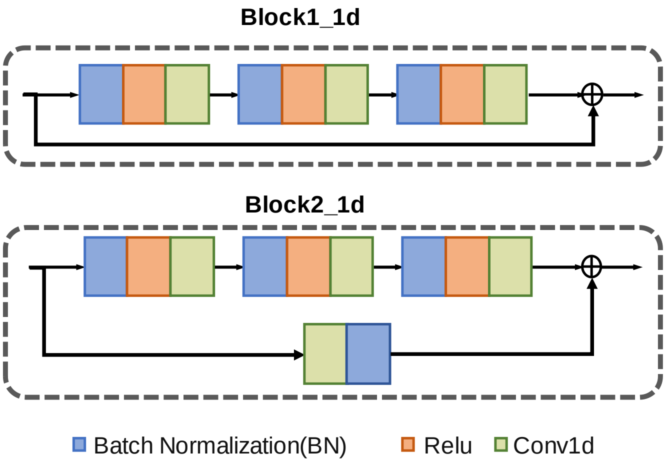

Supplementary Figure 5: CNN and Residual block.

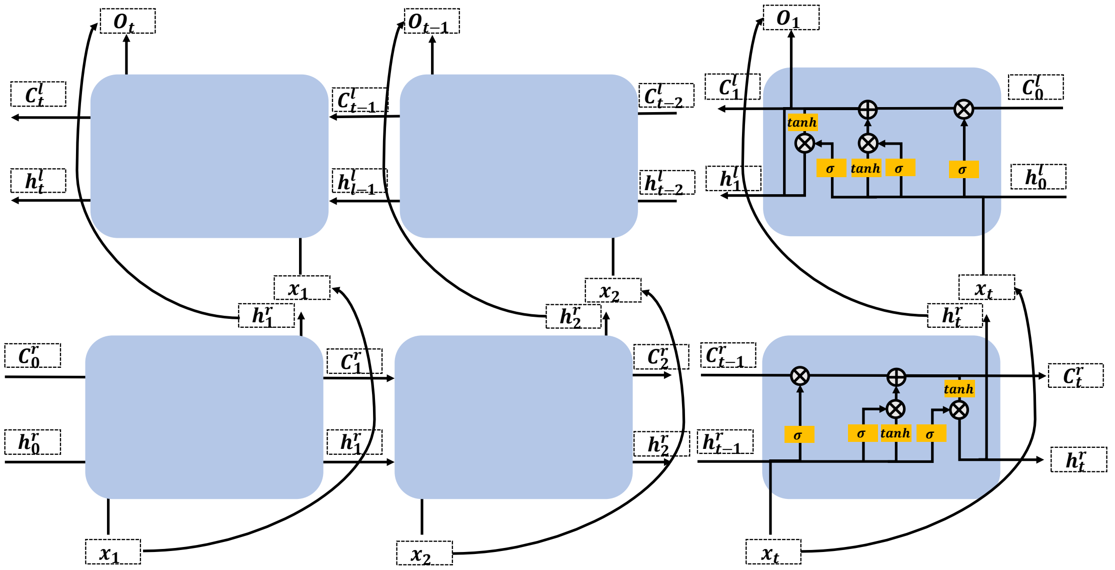

Supplementary Figure 6: BiLSTM schematic diagram.

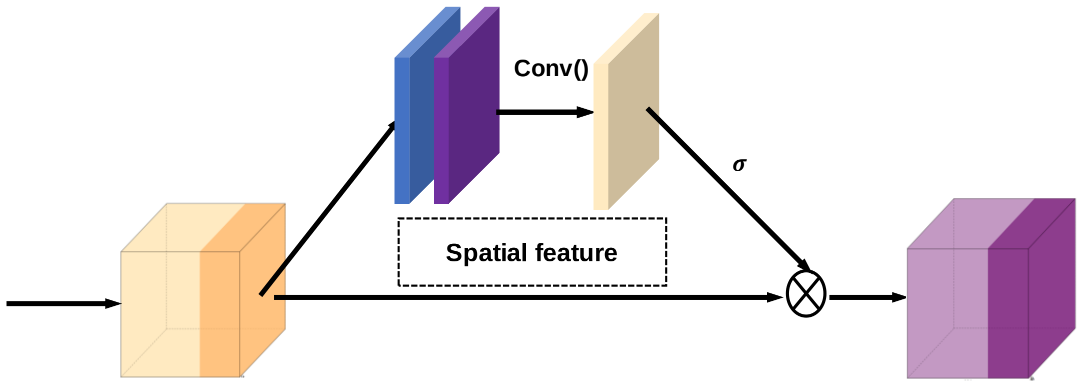

Supplementary Figure 7: ATT layer.

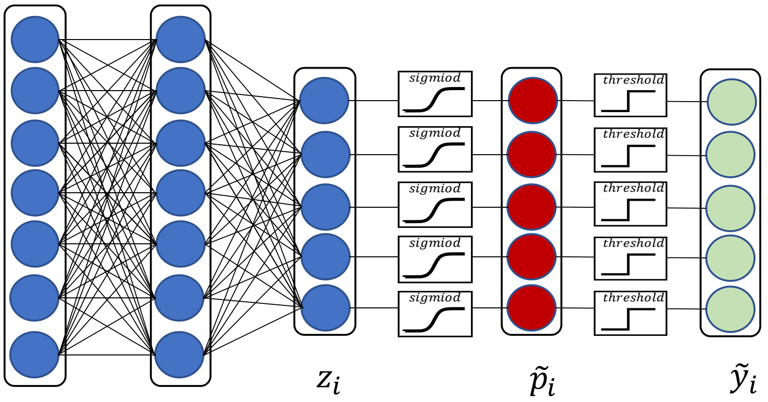

Supplementary Figure 8: Multi-label classifier.

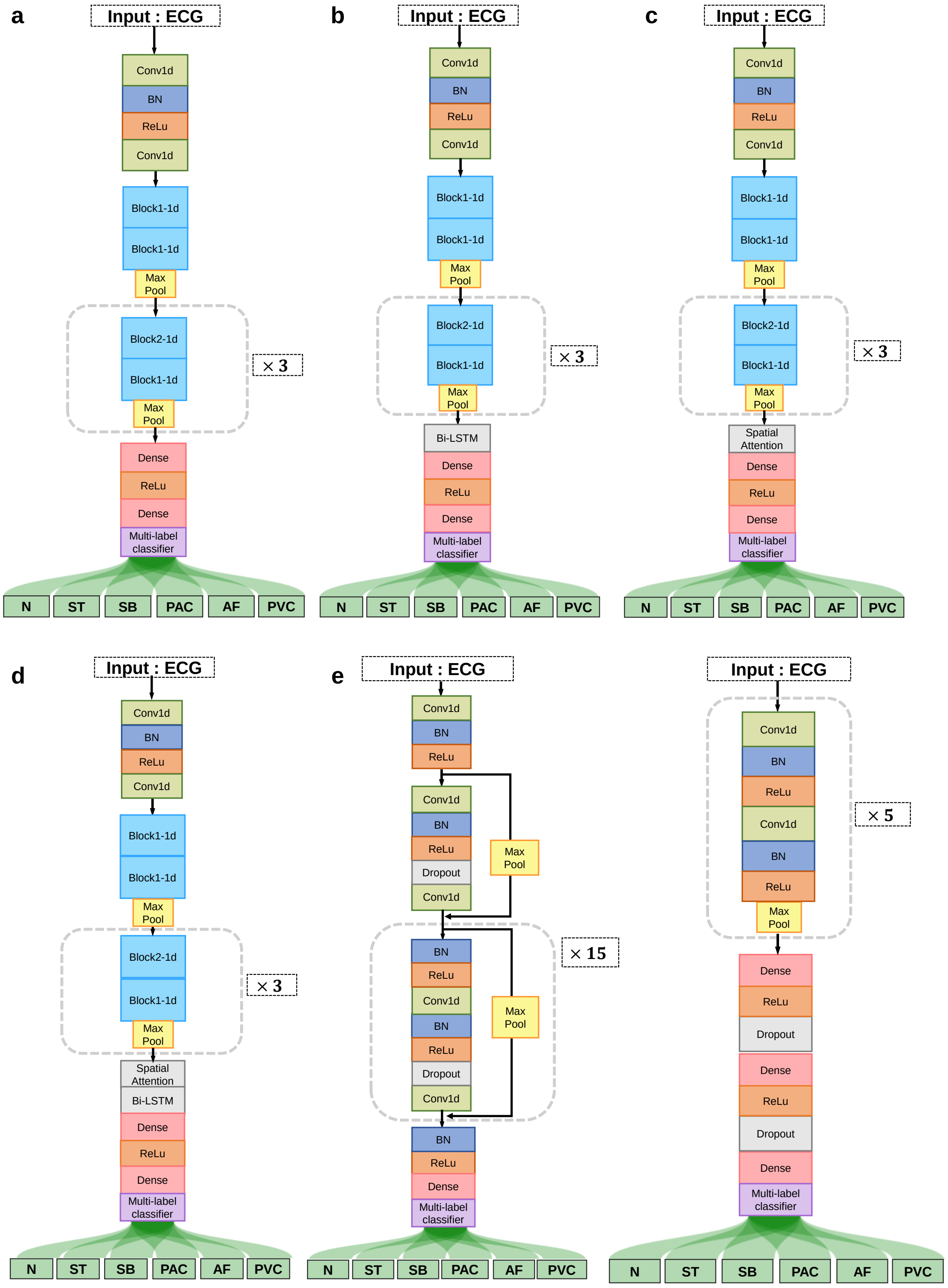

Supplementary Figure 9: Ablation experiments and comparison experiments models. a Ablation experiment without BiLSTM and ATT. **b** Ablation experiment without ATT. **c** Ablation experiment without BiLSTM. **d** Comparison experiments: The proposed method without medical post-processing module. **e** Comparison experiments: Ribeiro's method. **f** Comparison experiments: Simonyan's method[18].

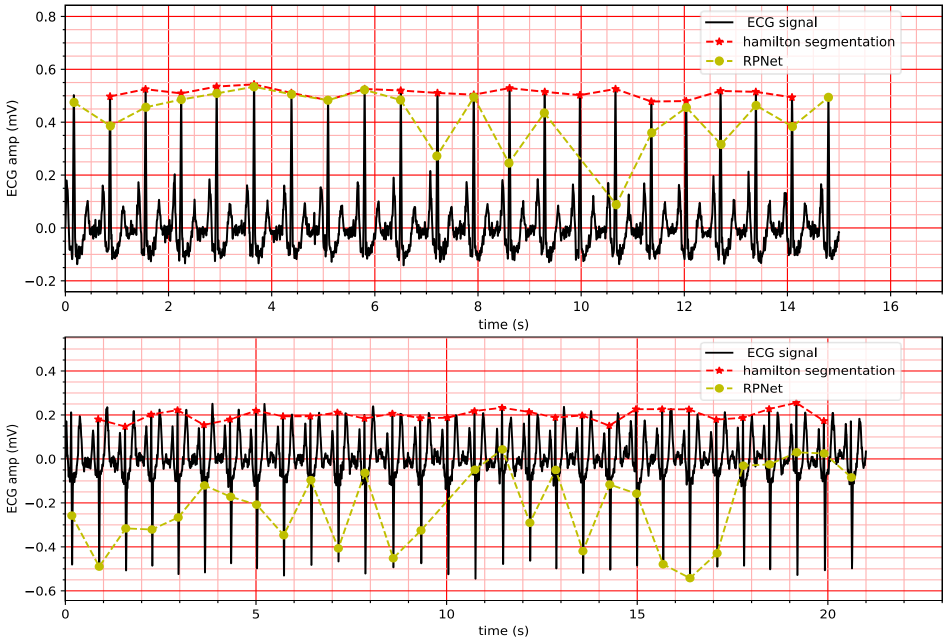

Supplementary Figure 10: **The visualization of two cases to show the differences between two algorithms**

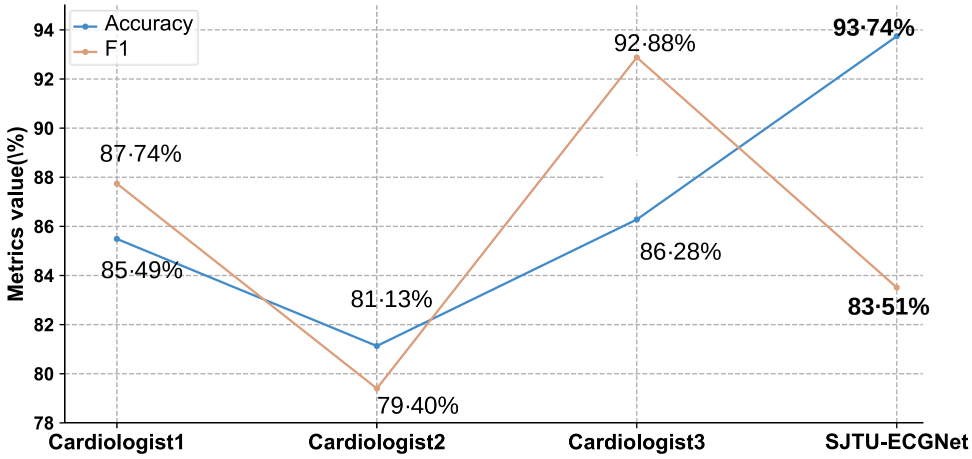

Supplementary Figure 11: Comparison of three cardiologists and SJTU-ECGNet in diagnostic accuracy and F1-macro in the re-label hidden set.

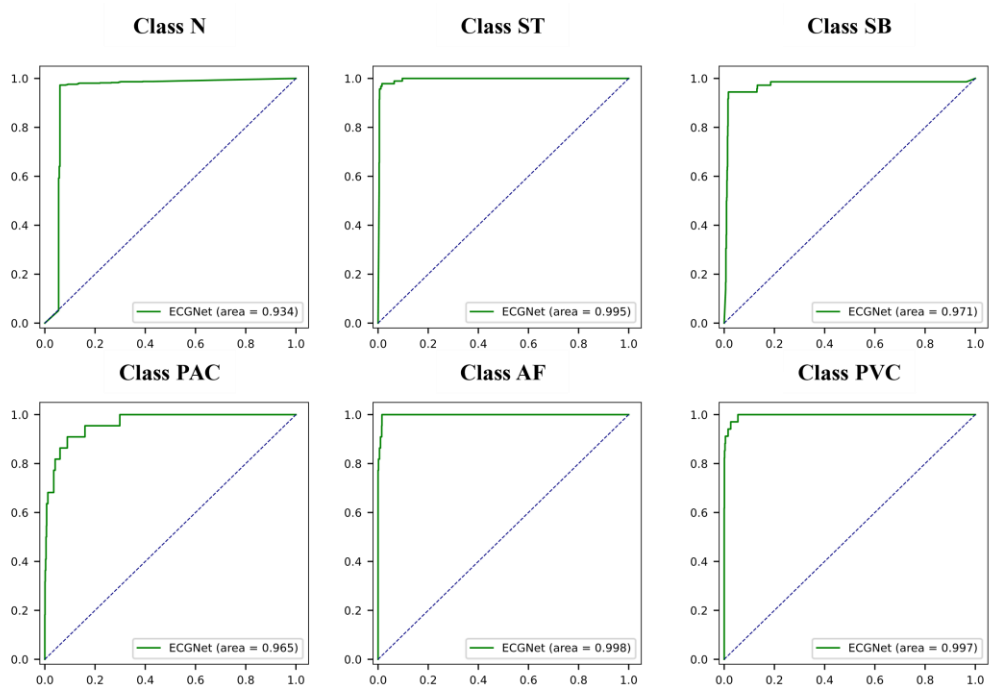

Supplementary Figure 12: ROC curves and AUC score of the deep learning model for 6 classification categories.

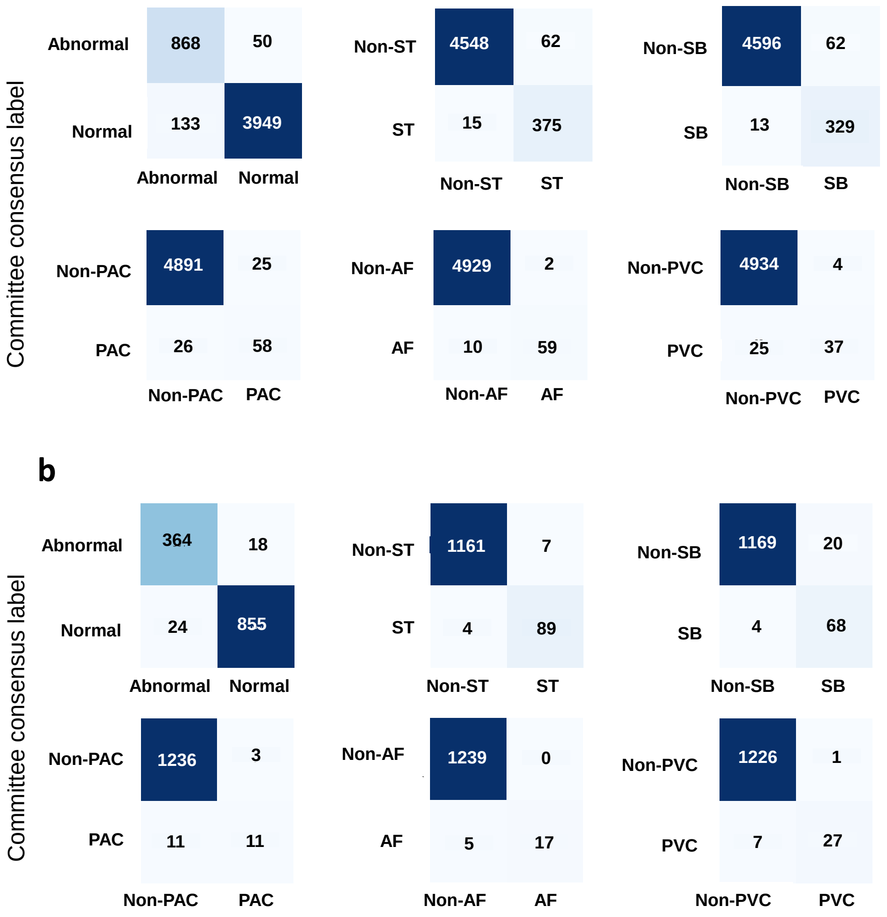

Supplementary Figure 13: Confusion matrices for different diseases. a Confusion matrices of test set. **b** Confusion matrices of hidden set. **(**ST=sinus tachycardia. SB= sinus bradycardia. PAC= atrial premature contraction. AF= atrial fibrillation. PVC= ventricular premature contraction.)

Supplementary References

1 S. Hong, W. Zhang, C. Sun, Y. Zhou, and H. Li. Practical Lessons on 12-Lead ECG Classification: Meta-Analysis of Methods From PhysioNet/Computing in Cardiology Challenge 2020. Frontiers in Physiology, 2021, 12: 811661.

2 Y. Bu, X. Cha, J. Zhu, Y. Su, D. Lai. Automatic detection model of hypertrophic cardiomyopathy based on deep convolutional neural network. Journal of Biomedical Engineering, 2022, 39(2): 285-292.

3 Fukushima K. Neocognitron: a self organizing neural network model for a mechanism of pattern recognition unaffected by shift in position. *Biol Cybern* 1980; **36**: 193–202.

4 Lecun Y, Bottou L, Bengio Y, Haffner P. Gradient-based learning applied to document recognition. *Proceedings of the IEEE* 1998; **86**: 2278–324.

5 Bonfanti S, Guerra R, Font-Clos F, Rayneau-Kirkhope D, Zapperi S. Automatic design of mechanical metamaterial actuators. *Nat Commun* 2020; **11**: 4162.

6 Long E, Lin H, Liu Z, *et al.* An artificial intelligence platform for the multihospital collaborative management of congenital cataracts. *Nat Biomed Eng* 2017; **1**: 1–8.

7 Jin Y, Qin C, Liu J, *et al*. A novel deep wavelet convolutional neural network for actual ECG signal denoising. Biomedical Signal Processing and Control 2024； **87**:105480.

8 Ulloa Cerna AE, Jing L, Good CW, *et al.* Deep-learning-assisted analysis of echocardiographic videos improves predictions of all-cause mortality. *Nat Biomed Eng* 2021; **5**: 546–54.

9 He K, Zhang X, Ren S, Sun J. Deep Residual Learning for Image Recognition. In: 2016 IEEE Conference on Computer Vision and Pattern Recognition (CVPR). 2016: 770–8.

10 Li D, Zhou J, Liu Y. Recurrent-neural-network-based unscented Kalman filter for estimating and compensating the random drift of MEMS gyroscopes in real time. *Mechanical Systems and Signal Processing* 2021; **147**: 107057.

11 Moyano J M, Gibaja E L, Cios K J, et al. Review of ensembles of multi-label classifiers: models, experimental study and prospects. Information Fusion, 2018, 44: 33-45.

12 S. Vijayarangan, V. R., B. Murugesan, P. S.P., J. Joseph and M. Sivaprakasam. RPnet: A Deep Learning approach for robust R Peak detection in noisy ECG. 2020 42nd Annual International Conference of the IEEE Engineering in Medicine & Biology Society (EMBC), Montreal, QC, Canada, 2020, pp. 345-348, doi: 10.1109/EMBC44109.2020.9176084.

13. Moody, G. B. & Mark, R. G. The impact of the MIT-BIH Arrhythmia Database. IEEE Engineering in Medicine and Biology Magazine 20, 45–50 (2001).

14. Ribeiro, A. H. et al. Automatic diagnosis of the 12-lead ECG using a deep neural network. Nat Commun 11, 1–9 (2020).

15. Ribeiro, A. H. et al. CODE-15%: a large scale annotated dataset of 12-lead ECGs (1.0.0) [Data set]. Zenodo. https://doi.org/10.5281/zenodo.4916206. (2021).

16. Wagner, P. et al. PTB-XL, a large publicly available electrocardiography dataset. Sci Data 7, 154 (2020).

17 Hamilton P. Open source ECG analysis. In: Computers in Cardiology. 2002: 101–4.

18 Simonyan K, Zisserman A. Very deep convolutional networks for large-scale image recognition. arXiv preprint arXiv:1409.1556, 2014.
