# Supplementary material for: Cardiologist-level interpretable knowledge-fused deep neural network for automatic arrhythmia diagnosis
Source: Commun Med (Lond). 2024 Feb 28;4:31. doi: 10.1038/s43856-024-00464-4 (PMC10901870; doi:10.1038/s43856-024-00464-4)
Supplement: Supplementary file 3 — Description of Additional Supplementary Files [file 43856_2024_464_MOESM3_ESM.pdf]

Description of Additional Supplementary Files

File Name: Supplementary Data 1
Description: The source data underlying Figure 2 and 3
