# Supplementary material for: Cardiologist-level interpretable knowledge-fused deep neural network for automatic arrhythmia diagnosis
Source: Commun Med (Lond). 2024 Feb 28;4:31. doi: 10.1038/s43856-024-00464-4 (PMC10901870; doi:10.1038/s43856-024-00464-4)
Supplement: Supplementary file 5 — Reporting summary [file 43856_2024_464_MOESM5_ESM.pdf]

nature portfolio

Corresponding author(s): Chengliang Liu
Last updated by author(s): Oct 8, 2023

Reporting Summary

Nature Portfolio wishes to improve the reproducibility of the work that we publish. This form provides structure for consistency and transparency in reporting. For further information on Nature Portfolio policies, see our Editorial Policies and the Editorial Policy Checklist.

Statistics

For all statistical analyses, confirm that the following items are present in the figure legend, table legend, main text, or Methods section.

n/a	Confirmed	
☐	☒	The exact sample size (*n*) for each experimental group/condition, given as a discrete number and unit of measurement
☐	☒	A statement on whether measurements were taken from distinct samples or whether the same sample was measured repeatedly
☐	☒	The statistical test(s) used AND whether they are one- or two-sided<br>*Only common tests should be described solely by name; describe more complex techniques in the Methods section.*
☒	☐	A description of all covariates tested
☒	☐	A description of any assumptions or corrections, such as tests of normality and adjustment for multiple comparisons
☒	☐	A full description of the statistical parameters including central tendency (e.g. means) or other basic estimates (e.g. regression coefficient) AND variation (e.g. standard deviation) or associated estimates of uncertainty (e.g. confidence intervals)
☒	☐	For null hypothesis testing, the test statistic (e.g. *F*, *t*, *r*) with confidence intervals, effect sizes, degrees of freedom and *P* value noted<br>*Give P values as exact values whenever suitable.*
☒	☐	For Bayesian analysis, information on the choice of priors and Markov chain Monte Carlo settings
☐	☒	For hierarchical and complex designs, identification of the appropriate level for tests and full reporting of outcomes
☒	☐	Estimates of effect sizes (e.g. Cohen's *d*, Pearson's *r*), indicating how they were calculated

Our web collection on statistics for biologists contains articles on many of the points above.

Software and code

Policy information about availability of computer code

Data collection	We developed two computer software to help data extraction and cardiologists re-labelling. As for data extraction software, we programed it by Python and used it to extract ECG recordings and labels from original clinical data. Then, we stored these ECG recordings and labels as HDF5 format for subsequent analysis. We developed a doctor-oriented web platform for cardiologists to label hidden dataset, as described in the manuscript. On this platform, ECG in hidden dataset was stored in a MySQL database and cardiologist checked each ECG recording and necessary information including age and sex. Cardiologists should choose several labels for each ECG. Finally, we collected these re-labelled data from MySQL database and stored them into HDF5 format for evaluation.
Data analysis	For data processing, we used python v3.8, pandas v1.2.4, numpy v1.18.5 and scipy v1.6.2.

For manuscripts utilizing custom algorithms or software that are central to the research but not yet described in published literature, software must be made available to editors and reviewers. We strongly encourage code deposition in a community repository (e.g. GitHub). See the Nature Portfolio guidelines for submitting code & software for further information.

Data

Policy information about availability of data

All manuscripts must include a data availability statement. This statement should provide the following information, where applicable:

- Accession codes, unique identifiers, or web links for publicly available datasets
- A description of any restrictions on data availability
- For clinical datasets or third party data, please ensure that the statement adheres to our policy

The main data supporting the results in this study are available within the paper and its Supplementary Information. The requests for training data will be reviewed by corresponding author to verify whether the request is subject to any intellectual property or confidentiality constraints. Requests for patient-related data not included in the paper will not be considered. Any data that can be shared will be released via a Material Transfer Agreement for non-commercial research purposes.

Human research participants

Policy information about studies involving human research participants and Sex and Gender in Research.

Reporting on sex and gender	The dataset comprises 51261 clinical recordings from over 50000 patients by carefully selecting from the whole LSCP-ECGDS, where 48.3% are male and 51.7% are female with ages covering the range from the young to the elder (7.5% below 25 years, 39.2% from 25 to 50 years, 45.9% from 50 to 75 years and 7.4% over 75 years).
Population characteristics	We collected samples from a Chinese clinical database.
Recruitment	We collected samples from a clinical database. The potential source of bias was the ECG of Chinese people in our geographic region.
Ethics oversight	This study was approved by Shanghai Jiao Tong University and Shanghai First People's Hospital Affiliated to Shanghai Jiao Tong University Review Board.

Note that full information on the approval of the study protocol must also be provided in the manuscript.

Field-specific reporting

Please select the one below that is the best fit for your research. If you are not sure, read the appropriate sections before making your selection.

☒ Life sciences ☐ Behavioural & social sciences ☐ Ecological, evolutionary & environmental sciences

For a reference copy of the document with all sections, see nature.com/documents/nr-reporting-summary-flat.pdf

Life sciences study design

All studies must disclose on these points even when the disclosure is negative.

Sample size	The dataset comprises 51261 clinical recordings from over 50000 patients by carefully selecting from the whole LSCP-ECGDS, where 48.3% are male and 51.7% are female with ages covering the range from the young to the elder (7.5% below 25 years, 39.2% from 25 to 50 years, 45.9% from 50 to 75 years and 7.4% over 75 years).
Data exclusions	The pre-established data exclusion criteria are: 1. ECG containing diseases other than the arrhythmias listed in this paper were excluded. 2. Exclude ECG with low signal quality. 3. ECG in 2017 was used for external test set to evaluate model due to different analytical format standards.
Replication	We contained all our software dependencies in a Github. All code is version-controlled and experiments can be reproduced on hardware with NVIDIA GTX 1080 GPU. Data used in this study was stored and backed up for future experimental reproducibility.
Randomization	The dataset was randomly divided in training set, verification set, test set and hidden set.
Blinding	The cardiologists were blinded from actual diagnosis results and from the model's performance, until the final release of the results.

Reporting for specific materials, systems and methods

We require information from authors about some types of materials, experimental systems and methods used in many studies. Here, indicate whether each material, system or method listed is relevant to your study. If you are not sure if a list item applies to your research, read the appropriate section before selecting a response.

Materials & experimental systems

n/a	Involved in the study
☒	☐ Antibodies
☒	☐ Eukaryotic cell lines
☒	☐ Palaeontology and archaeology
☒	☐ Animals and other organisms
☒	☐ Clinical data
☒	☐ Dual use research of concern

Methods

n/a	Involved in the study
☒	☐ ChIP-seq
☒	☐ Flow cytometry
☒	☐ MRI-based neuroimaging
